# Effect of gastro-intestinal nematode infection on sheep performance: a systematic review and meta-analysis

**DOI:** 10.1186/s13071-015-1164-z

**Published:** 2015-10-24

**Authors:** Fabien Mavrot, Hubertus Hertzberg, Paul Torgerson

**Affiliations:** Section for Veterinary Epidemiology, Vetsuisse Faculty, University of Zurich, Zurich, Switzerland; Institute of Parasitology, University of Zurich, Zurich, Switzerland

**Keywords:** Sheep, Gastro-intestinal nematodes, Impact, Weight, Wool, Milk, Production

## Abstract

**Background:**

Gastrointestinal nematode (GIN) infections are common in domestic sheep and impact directly and indirectly on the health of infected animals as well as on the associated economic production. In this study, we aim at summarizing the current knowledge on the influence of GIN infections on sheep production by conducting a systematic review. A subsequent meta-analysis of relevant studies was performed to provide an estimate of the effect of GIN infections on weight gain, wool production and milk yield.

**Methods:**

A literature search was performed on the CAB, Pubmed and Web of Science database for the period 1960–2012. Inclusion criteria were: 1) Measurement of at least one production parameter. 2) Comparison between groups of sheep with different nematode burdens. 3) Same conditions regarding all aspects except parasite burden between groups. 4) Quantitative measurements of one or more production traits.

**Results:**

Altogether, 88 studies describing 218 trials were included in this review. The majority of studies (86 %) reported that GIN infections had a negative effect on production but this was reported to be statistically significant in only 43 % of the studies. Meta-analysis indicated that performances of sheep infected with nematodes was 85, 90 and 78 % of the performance in uninfected individuals for weight gain, wool production and milk yield respectively. Our results suggest a possible reporting bias or small study effect for the estimation of the impact of GIN infections on weight gain. Finally, a general linear model provided an estimate for the decrease in weight gain in relation to the increase in faecal egg count of nematodes.

**Conclusion:**

This study underlines the importance of GIN infections for sheep production and highlights the need to improve parasite management in sheep, in particular in face of challenges such as anthelmintic resistance.

**Electronic supplementary material:**

The online version of this article (doi:10.1186/s13071-015-1164-z) contains supplementary material, which is available to authorized users.

## Background

Gastro-intestinal parasitism is one of the most common infections in livestock. Clinical signs and sequelae are dependent on the parasite fauna present and the intensity of infection. In sheep, these can range from subclinical weight loss to lethal pathologies such as anaemia, diarrhoea and severe protein loss [1]. In addition, parasitism can have indirect consequences on metabolism such as mobilisation of proteins for an immune-response, reduced feed intake due to anorexia or increased susceptibility to other pathogens [2–4]. Since the 1960s the use of anthelmintics has become an important strategy to control nematode infections in livestock and increase their production performance [5]. For example, Sanchez et al. [6] reported the results of a meta-analysis which concluded that dairy cattle gained an estimated increase in milk production of 0.35 kg/day following treatment against gastro-intestinal nematodes.

According to the Food and Agriculture Organisation [7] the sheep population amounted to 1.2 billion in 2012, distributed as follow: Asia, 44.9, Africa, 27.6, Europe, 11.1, Oceania, 9.1 and Americas, 7.3 %. Worldwide, sheep production for 2012 was 10 million tons of milk, 8 million tons of meat and 2 million tons of wool. Distribution of meat production is correlated with distribution of sheep population whereas milk production is mainly based in the Mediterranean region and the Near East and wool production is proportionally more important in Oceania and Asia [7, 8].

Sheep represent an important source of income in many countries [8, 9] and although the effects of parasitism on production have been recognized [10], there is still a need to quantify these losses. Anthelmintic resistance and climate change is likely to alter the geographical distribution of parasites and their impact on production animals, thus increasing the need for a clear understanding of the cost of parasitism in order to develop sustainable control strategies [10, 11].

Systematic reviews and meta-analysis have been widely used to summarize results of different studies made on one particular subject. The increased sample size obtained when combining studies as well as the possibility to identify error sources such as publication bias improve the quality of the analysis and strengthen its conclusions. In particular, in medical research, those methods are frequently used to measure the efficacy of a treatment or assess the relationship between risk factors and a medical condition [12].

Here we undertake a systematic review to identify studies which evaluated the impact of gastrointestinal nematodes on different aspects of sheep production and summarize their results. Meta-analysis was then applied to the data in suitable studies to evaluate the effect of gastrointestinal nematode (GIN) infections in sheep on weight gain, wool production and milk yield, which are the main economic purposes of sheep breeding [9, 13]. Finally, since effects of parasitism are expected to depend on the parasite burden [10], we also analysed the relation between quantitative egg excretion (used as a proxy for parasite burden in young animals [14]) and production performance.

## Methods

The methodology followed the “Preferred Reporting Items for Systematic Reviews and Meta-Analyses” (PRISMA, [15]) recommendations for improving the standards of meta-analyses. A PRISMA check list is provided as supplementary material to this publication (see Additional file 1). Statistical analysis and figures were made using the R statistical program [16].

### Search strategy

The databases CAB, Pubmed and Web of Science were searched for the period 1960–2012 in order to retrieve relevant studies. Three production traits were taken in consideration: weight gain, milk yield and wool production. Searches were performed using different key words distributed among three search terms: [nematode/parasite/anthelmintic/parasite control] AND [weight/growth/wool/fleece/milk/production] AND [sheep]. All possible combinations of the three terms were used (e.g., anthelmintic AND fleece AND sheep).

### Inclusion and exclusion criteria

Studies were first screened by scanning the title and abstract. Suitable studies were retained for more detailed examination. Studies were then selected for inclusion if they met the following criteria:

1. A production parameter was measured (weight gain in lambs, wool production or milk yield).
2. There were at least two groups of sheep which differed in their gastro-intestinal nematode burden (e.g., infected sheep group vs control or dewormed group vs control).
3. There were no other reported differences between the groups (e.g., feeding, breed, housing, age, infection with trematodes).
4. The report quantified the production of each group or whether there was a significant difference between groups.

For studies describing more than one trial, each trial was included separately in the review. Additionally, for studies where more than one group were compared to the control group, each group being compared with the control group was considered as a separate trial. Finally, for studies measuring more than one production trait, the recorded gain in each production trait was considered as a separate trial.

Trials were classified into two categories:

- Infection/control trials: trials with an infected group (INF) and a control group (CONT) with no or a negligible nematode infection (animals raised and kept in a nematode-free environment or regularly treated and with a mean faecal egg count (FEC) < 50 eggs per gram (EPG) determined by repeated measurements over the trial’s duration).
- Burden trial: Trials which compared production between two groups of nematode infected sheep but in which one group had a high parasite burden (HPAR) and the other group had a lower burden (LPAR).

Subsequently, only trials of the type infection/control were included in a meta-analysis of the effect of infection status on performance. In addition an analysis on the effect of nematode burden on performance was undertaken using all trials (infection/control and burden types) for which FEC was monitored in every group (based on repeated measurements over the duration of the trial).

### Effect of infection status on performance

Using the meta and metafor packages in R [17, 18], a meta-analysis was undertaken to evaluate the effect of infection status on production. To construct a confidence interval around the final gain in production, only trials reporting a standard error of the measured outcome were included in the analysis.

A standardized measurement of the gain in production was obtained by computing the ratio of the performance in the INF group over the performance in the control group (no parasite burden). This allowed comparison between different studies, since the reported performance (in grams of body weight/fleece or litres milk) can be influenced by other factors such as breed, feeding or trial duration or was measured with different units between different studies (e.g., wool production measured either in grams of wool at shearing or in mm wool growth).

Since this standardized measurement is a ratio, logarithmic transformation, as described in [19], was used for the computation of confidence intervals and to perform further analysis.

Analysis was performed separately for the three production traits (weight gain, wool production and milk yield) as well as for the type of nematode infection: either mixed species infection or mono-infection with *Haemonchus contortus*, *Trichostrongylus colubriformis* or *Teladorsagia (Ostertagia) circumcincta*. Additionally, only studies performed on growing animals less than one year old were included in analysis measuring weight gain.

Linear regression test for funnel plot asymmetry [20] was conducted to control for publication bias or small-study effect and the fill-and-trim method [21] was used to compute an adjusted estimate of the overall effect when needed.

### Relation between egg excretion and performance

We built a generalized linear model (GLM) to estimate the impact on production in relation to the faecal nematode egg output. The measured outcome was defined as the log-transformed ratio of production of the infected group over the control. In addition to the log-transformed difference in mean FEC between the groups five additional explanatory variables were included in the model: 1) the absolute value of the latitude at which the trial was conducted (ranging from 0 at the equator to 90 at each pole) which served as proxy for a possible effect of climate [22, 23]; 2) trial duration in weeks, since the impact of a pathogen might not only depend on infection intensity, but also on infection duration [24] or development of immunity by the host [2]; 3) age classes of the animals (1–6 months or 7–12 months) since effect of parasitism and host response can vary with the age of the lambs [25, 26];4) study design (infected vs control, treated vs untreated or other) was added as a predictive variable since infection pressure and its fluctuation over the trial duration might differ between the different type of trials. In addition, infection course and host response might differ between experimentally or naturally acquired parasite infection [27]; 5) FEC diagnostic method (gravitational or centrifugal) was also included since it might influence the estimate of parasite burden in animals [28, 29]. Additionally, trials were assigned weight in the model according to their sample size. The model was constructed using backward selection based on the Aikake Informaton Criterion (AIC).

Similarly to the meta-analysis on the effect of infection status, we considered trials separately, depending on the three production traits measured as well as on the species of nematodes infecting the animals. However only trials measuring weight gain in lambs with mixed parasite infection were in sufficient quantity to provide a robust model (*n* = 73) and thus, only those trials were used for modelling. Finally, we also investigated the relationship between FEC and nematode burden in studies which necropsied animals and performed a worm count of the whole gastrointestinal tract.

## Results

Searching the three databases, a total of 45,402 results corresponding to 11,873 studies were obtained. Of these, 265 studies remained after an initial screening of titles and abstracts. Finally 85 studies were included following full paper review. The main reasons for excluding studies were: study on agent other than nematodes, study on species other than sheep, production parameters of interest not measured and difference between the experimental groups regarding aspects other than parasite burden (e.g., food, breed). During this process, three additional studies were identified from the cited references of screened studies and also included in the review resulting in a total of 88 studies [30–117].

These 88 studies described a total of 218 trials. Twenty-two studies described only one trial. The other 66 studies included at least two trials. Mean sample size in the trials was 49 (median: 20, range: 8–500) and average trial duration was 16 weeks. Gain in production was assessed by treating animals with anthelmintics in 42 studies, through experimental infection in 40 studies, through different pasture management methods (e.g., pasture rotation) in five studies and by comparing animals with naturally high and low FEC in one study. Studies originated from 23 different countries. The United-Kingdom and Australia were the countries with the most studies (18 and 12, respectively) and account for more than one third of the total studies included in this review (Table 1).

**Table 1:** Country of origin of 88 studies assessing impact of parasitism on production traits of sheep

Europe (38)	*n*	Oceania (21)	*n*	Americas (14)	*n*	Africa (10)	*n*	Asia (5)	*n*
UK	18	Australia	12	Brazil	7	Kenya	5	India	1
Spain	5	New-Zealand	9	USA	4	Ethiopia	2	Indonesia	1
Greece	4			Argentina	1	South-Africa	2	Iraq	1
Italy	4			Mexico	1	Nigeria	1	Malaysia	1
France	3			Venezuela	1			Pakistan	1
Denmark	1								
Germany	1								
Ireland	1								
Switzerland	1								

Table 2 shows a summary of the reported effect of parasitism on production in sheep. Altogether, 187 trials (85.8 %) reported a negative effect of nematode infection on production, with 94 (43.1 %) of them reporting a statistically significant effect. In contrast, a positive effect of parasitism on production was found in 24 trials (10.9 %) and seven (3.2 %) trials reported no differences in production between parasitised and control animals.

**Table 2:** Effect of gastro-intestinal nematode infection on production in sheep reported in 218 trials

Reported effect of parasitism on production	Measured production trait	Total number of trials	Number of trials reporting a p-value	Number of statistically significant trials (p < 0.05)	% of statistically significant trials
Negative	Weight gain	147	122	74	60.66
	Wool growth	24	21	11	52.38
	Milk yield	16	16	9	56.25
	Total	187	159	94	59.12
Positive	Weight gain	18	15	2	13.33
	Wool growth	4	1	0	0.00
	Milk yield	2	2	0	0.00
	Total	24	18	2	11.11
None	Weight gain	6	6	0	0.00
	Wool growth	1	0	0	0.00
	Milk yield	0	0	0	0.00
	Total	7	6	0	0.00

Altogether, statistical testing of the effect of parasitism on production was reported in 183/218 trials. There was no significant difference in the proportion of trials reporting a p-value between trials describing a negative effect of parasitism and those reporting a positive effect (159/187 vs 18/24, Fisher exact test: *p* = 0.237).

However, a larger proportion of trials reported a significant negative effect of parasitism compared to trials reporting a significant positive effect (94/159 vs 2/18, Fisher exact test: *p* < 0.001).

### Effect of infection status on performance

A total of 94 trials were of the type infection/control and met requirements to be included in the meta-analysis (70 trials measuring weight gain, 5 trials measuring milk yield and 19 trials measuring wool production).

In 78/94 trials, a negative effect of parasitism on production was reported (weight gain: 59/70, milk yield: 5/5, wool production: 14/19). However, in 14 trials (weight gain: 10/70, wool production: 4/19) parasitism was associated with an increased performance. Finally, in two trials (one measuring weight gain and one measuring wool production), the authors reported there were no differences between infected and control animals.

**Fig. 1:**
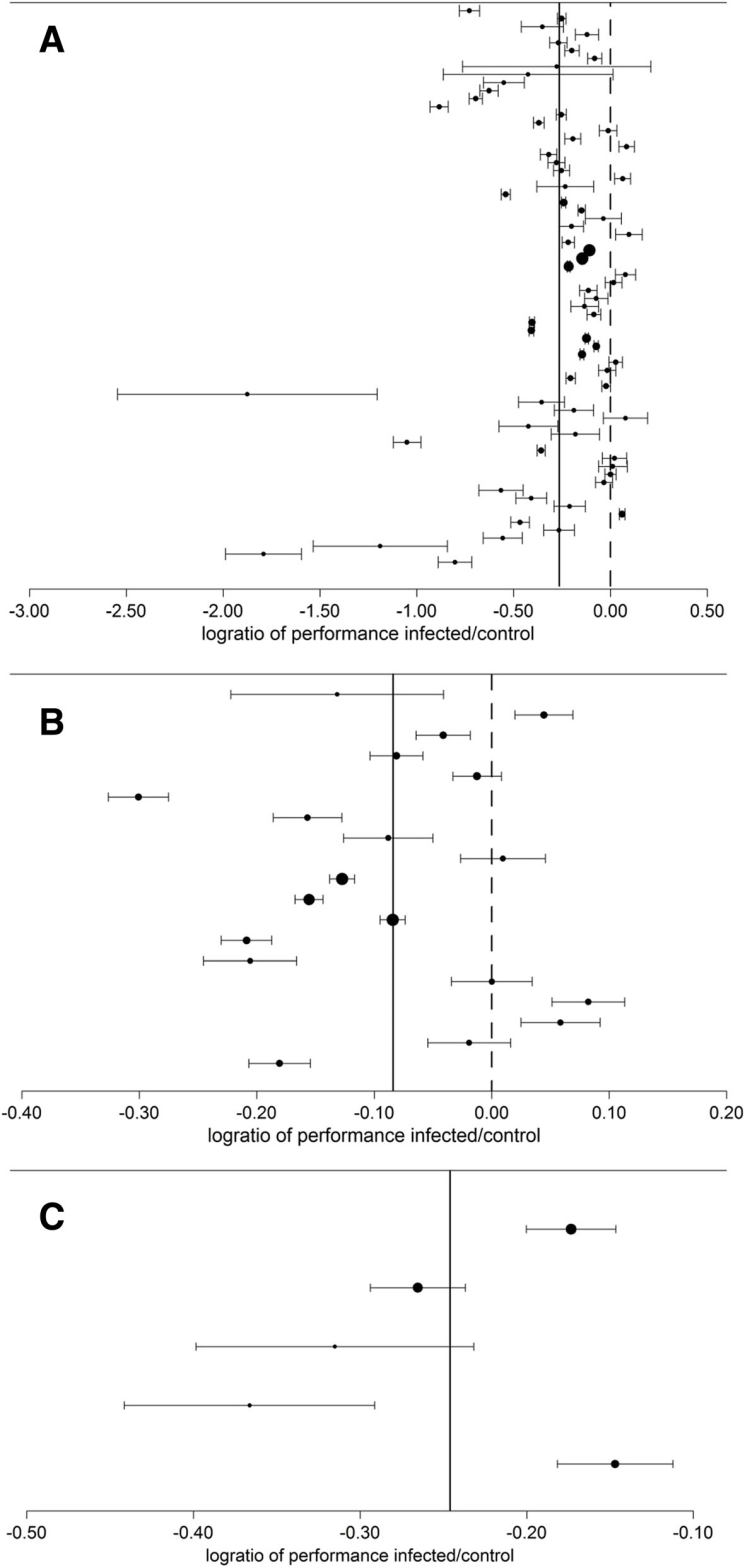
Forest plots of 94 trials included in the meta-analysis of impact of gastro-intestinal nematode infection on weight gain (**a**, *n* = 70), wool production (**b**, *n* = 19) and milk yield (**c**, *n* = 5) in sheep. Black dots represent the log-transformed ratio of performance of the infected over the control group in each trial. Dot sizes are proportional to the sample sizes in the trial and horizontal bars give the standard error of the estimate. Vertical dotted lines indicate the zero (no effect of nematode infection on production) and vertical continuous lines show the overall estimate for all the trials in each performance trait

**Fig. 2:**
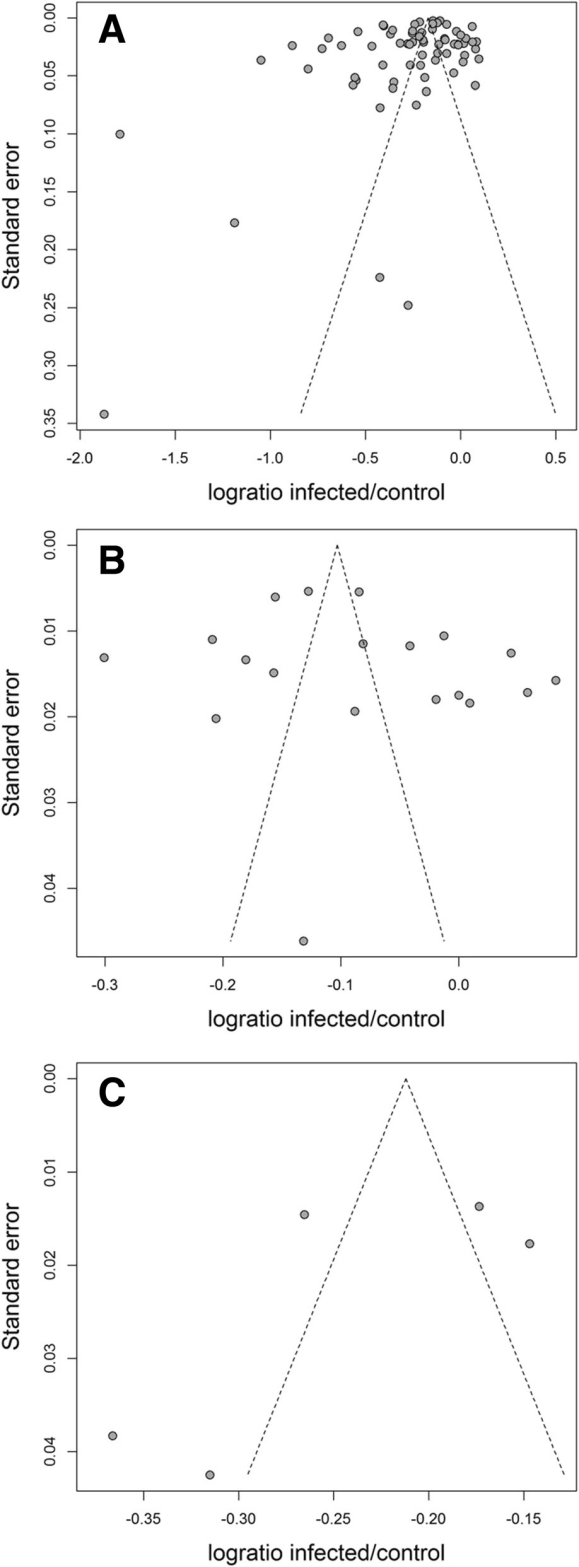
Funnel plots with 95 % pseudo-confidence limits of 94 trials included in the meta-analysis of impact of nematodes on weight gain (**a**, *n* = 70), wool production (**b**, *n* = 19) and milk yield (**c**, *n* = 5) in sheep. Treatment effect (log-transformed ratio of performance of infected over control animals) is given on the X-axis and standard error of the estimate is represented on the Y-axis

Results of the meta-analysis are summarized in Fig. 1 and Table 3. Test for funnel plot asymmetry indicated a possible bias for trials reporting weight gain (*p* = 0.032) but not for wool production (*p* = 0.307) and milk yield (*p* = 0.336). Fig. 2 shows the funnel plots for the three production traits.

**Table 3:** Meta-analysis of 94 trials on the estimated effect of parasitic infection on sheep performance

Production trait	Infection type	Number of trials	Ratio production infected/control	*95 % C.I.*	Number of trials reporting egg excretion	Mean number of eggs per gram faeces in infected animals
Weight gain	Mixed species^a^	30	0.74	0.71–0.77	22	2336
Weight gain	*H. contortus*	20	0.79	0.71–0.87	20	4019
Weight gain	*T. colubriformis*	12	0.78	0.71–0.87	12	1070
Weight gain	*T. circumcincta*	8	0.81	0.66–0.99	4	296
Wool production	Mixed species^a^	14	0.9	0.86–0.93	11	3788
Wool production	*H. contortus*	2	1.04	0.96–1.13	2	7585
Wool production	*T. colubriformis*	2	1.02	0.95–1.1	2	1359
Wool production	*T. circumcincta*	1	0.83	0.81–0.86	1	201
Milk yield	Mixed species^a^	5	0.78	0.73–0.84	1	527

^a^Main species were of the genus Haemonchus, Teladorsagia, Trichostrongylus, Cooperia and Nematodirus

Altogether, estimates for the production ratio of infected animals over control were:

- 0.77 (95 % CI: 0.74–0.79) for weight gain or 0.85 (95 % CI: 0.82–0.88) after adjustment for reporting bias,
- 0.90 (95 % CI: 0.86–0.93) for wool production,
- 0.78 (95 % CI: 0.73–0.84) for milk yield.

In 75 trials, mean FEC over trial duration were reported for the infected group and ranged from 100 to 12,000 EPG (for details see table 3).

### Relation between parasite excretion and performance

**Fig. 3:**
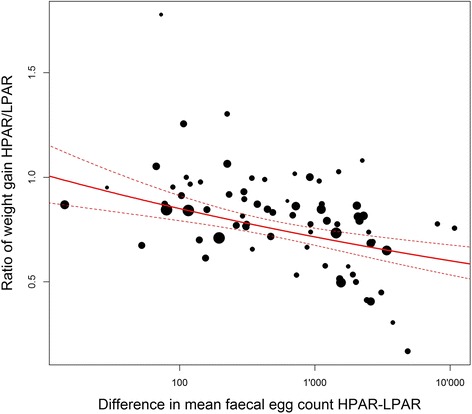
Decrease in weight gain of sheep by increasing infection level with mixed species of gastrointestinal nematodes. Mean difference in faecal egg counts between low parasite burden animals (LPAR) and high parasite burden animals (HPAR) is used as an indicator of level of infection and shown on the X-axis. Y-axis shows the ratio of weight gain of HPAR over LPAR. The continuous line shows the estimated effect of nematode infection with a 95 % confidence interval (dotted lines) computed with a Generalized Linear Model using the results of 73 trials (black dots with sizes proportional to sample size of the trials)

The best model (AIC: 27.532) included only increases in FEC as a predictor of the weight gain ratio between HPAR and LPAR groups (21.37 % of deviance explained). Fig. 3 shows the observed effect of parasitism recorded in the trials and the estimate of the model.

Altogether, by mixed species infection, an increase in FEC of 100, 1’000 and 10’000 EPG resulted in the HPAR lambs gaining 0.85, 0.71 and 0.6 times the weight of the LPAR lambs, respectively).

Finally, in 9 studies, lambs from either the HPAR groups or both HPAR and LPAR groups were necropsied and worm counts of the whole gastrointestinal tracts were performed. Altogether, worm count ranged from 30 to 41’718 and there was a positive correlation between mean FEC before slaughter and worm count (*n* = 26, spearman’s rho = 0.71, *p* < 0.001).

## Discussion

In this systematic review, a number of studies describing the relation between parasite infection and production in sheep were identified. The large majority of studies focused on the effect of parasitism on weight gain and relatively few studies measured other parameters such as wool production or milk yield.

Altogether, although the large majority of the trials reported a negative effect of parasitism on production, only 58.3 % of the trials for which a p-value was provided found this effect to be statistically significant. This lack of statistical significance could be due to the relatively small sample size in many of the studies as the median sample size in all the studies included in this review was only 20.

When looking at the trials comparing parasite-free and infected animals, the results of the meta-analysis indicate that, in parasite infected animals, the production in terms of weight gain, wool, and milk is respectively 77, 90 and 78 % of the production of parasite-free animals. Analysing the separate impact of different species of nematodes gave similar estimates, with wool production being less influenced than weight gain by parasitism.

Testing for funnel plot asymmetry indicated that trials measuring weight gain were probably biased. Therefore the adjusted estimate of infected animals gaining 85 % of the weight of non-infected animals seems more reliable than the 77 % unadjusted estimate.

In contrast, no bias was detected following the meta-analyses of trials measuring wool production and milk yield. However, testing for bias is unreliable when the meta-analysis includes a small number of studies [118]. Thus there is the possibility of bias in the estimates of the effect of parasitism on wool production and milk yield presented in this review. If that is the case, it is likely that, similarly to weight gain, our analysis overestimates the true impact of parasitism on those production traits.

Nevertheless, our results indicate that milk yield and weight gain are much more influenced by parasitism than wool production. Coop et al. [2] proposed that sheep respond to parasitism by shifting resource allocation with higher priority to maintaining vital body function, with other function such as weight gain and lactation being given a lower priority, and thus more likely to receive less resources in case of parasitism. It is possible that wool growth is part of sheep vital functions, which might explain the smaller effect of parasitism on this parameter.

In a review of the effect of parasitism in dairy cow production, Sanchez et al. [6] noted that level of parasitic infection is likely to be an important factor determining the effect on the milk yield and probably accountable for the large variation of the effect reported in the different studies. Similarly, only a minority of the studies included in the present review reported a level of infection, either by describing the initial parasite dose in case of experimental infection trials or by sacrificing animals to perform a post-mortem worm count.

In another meta-analysis, Kipper et al. [119] estimated that parasite-infected pigs had a daily weight gain 31 % inferior than non-infected individuals. Kipper did not discriminate between the different species of parasite when estimating their impact. He argued that the main effect of parasitism was due to the host adaptation to an infection and its immune response rather than to the species involved. The present study seems to support this argument since the estimate of the impact of the different nematode species considered separately were quite similar to the overall estimates for each production trait. However, because of the small number of trials for each separate nematode species, those estimates have to be interpreted with caution.

While FEC is usually considered a reliable indicator of nematode burden in small ruminants [14, 72, 120], some authors pointed out that the relationship between both variables might be more complex and involves other factors such as parasite density and diversity [121, 122] or host age and development of immunity [123, 124]. In this review, we found a strong relationship between FEC at slaughter and gastrointestinal worm count in lambs. It must be noted, though that those were averaged values which did not allow to account for individual variability and that the amount of groups for which worm count was reported was small (*n* = 26).

In the GLM presented here, increase in FEC was the only variable included in the best model. It was significantly associated with a decrease in weight gain and explained 21 % of the total deviance. None of the other variables tested in the analysis were selected in the final model. However, because of a strong heterogeneity and a lack of precise information in the included studies, we summarized the variables study design and FEC diagnostic method into two or three rough categories (e.g., gravitational *vs* centrifugal). This simplification might limit the ability of the model to detect an effect for those variables. For the same reason, other potentially relevant predictors such as breed, diet or co-infection with other pathogens could not be included in the analysis.

Although, alternative indicator such as plasma antibodies or pepsinogen level have been proposed [125], the results of this review corroborate that FEC can help evaluate nematode burden and its impact on weight gain in lambs. Additionally, procedures requiring blood sampling of individuals is more expensive in term of time and resources than FEC which make them less attractive for monitoring purpose. However, other less invasive parameters such as body condition or FAMACHA scores have proven themselves helpful in the frame of targeted selective treatments [126] and should be further propagated.

Finally, most of the studies identified with naturally infected animals used classical anthelmintic compounds in their experimental design. Although the efficacy of such products is widely acknowledged, increasing resistance of GIN to anthelmintics is reported worldwide [127, 128]. This review demonstrates that an increase in non-responsiveness to classical anthelmintics will have an important impact on sheep production and underlines the need for alternatives to chemical worm control such as pasture management, resistant breed or vaccination [129].

## Conclusion

This study confirms the importance of GIN infections on sheep performance and underlines the advantages of parasite control in production animals. The consequences of GIN infections seem to be similar for different species of parasites but seem to influence milk yield and weight gain more than wool production.

## Additional file

Additional file 1:
**PRISMA 2009 Checklist.** (Docx 30 KB)
